# Supplementary material for: Deletion of wfs1 Impairs Oligodendrocyte Precursor Cells Dorsal Distribution and Myelination Through the wfs1-hmgcs1 Axis in Zebrafish
Source: Biology (Basel). 2026 Aug 16;15(16):1402. doi: 10.3390/biology15161402 (PMC13510151; doi:10.3390/biology15161402)
Supplement: Supplementary file 1 [file biology-15-01402-s001.zip › File S1-Transcriptome sequencing data and differentially expressed genes (DEGs) in wfs1-- zebrafish at 5 dpf/2.KEGG/wfs1KOvsWT/ALL/wfs1KOvsWT.all_KEGGenrich.html]

Pathway Enrichment


# The most enriched pathway terms

Statistic method: hypergeometric test

FDR correction method: Benjamini and Hochberg

| Term | Description | Sample number | Background number | P-value | Corrected P-value | Gene\_id | Gene\_name | KEGG\_ID |
| --- | --- | --- | --- | --- | --- | --- | --- | --- |
| dre00590 | Arachidonic acid metabolism | 18/900 | 72/8379 | 0.000455771650076192 | 0.0430928439895198 | 406449\_1 406449 494108 767759 559020 406370\_1 494108\_1 393297 406370 566996 406575\_1 393592 797309 566996\_1 560649 399485 767688 100148818 | ptges3a ptges3a ptgr1 zgc:153704 ptgs2b pla2g6 ptgr1 zgc:56585 pla2g6 si:dkey-11k2.7 lta4h cyp2p9 cyp2j20 si:dkey-11k2.7 si:ch211-214p16.3 cyp2p10 zgc:154040 si:ch73-55i23.1 | dre:406449 dre:406449 dre:494108 dre:767759 dre:559020 dre:406370 dre:494108 dre:393297 dre:406370 dre:566996 dre:406575 dre:393592 dre:797309 dre:566996 dre:560649 dre:399485 dre:767688 dre:100148818 |
| dre00270 | Cysteine and methionine metabolism | 18/900 | 73/8379 | 0.000545479037842022 | 0.0430928439895198 | 335715 334195\_1 103911233 317744\_1 550524\_1 571201 561410\_1 791167 431773 445818 406688 431773\_1 30659\_1 103911241 563642 450084\_1 334195 678634 | mdh1ab mat1a LOC103911233 dnmt3bb.1 cbsa si:ch73-97h19.2 tat zgc:158222 enoph1 cthl got2a enoph1 dnmt3bb.2 LOC103911241 sdsl gss mat1a mat2al | dre:335715 dre:334195 dre:103911233 dre:317744 dre:550524 dre:571201 dre:561410 dre:791167 dre:431773 dre:445818 dre:406688 dre:431773 dre:30659 dre:571201 dre:563642 dre:450084 dre:334195 dre:678634 |
| dre04672 | Intestinal immune network for IgA production | 11/900 | 37/8379 | 0.00127081200693243 | 0.0562322212284064 | 799354 799354\_1 30762 368878 100006078\_1 30763 571282 100006078 797491\_1 100151365\_1 100333914 | cxcl12b cxcl12b mhc2dab si:busm1-266f07.2 ccr9b mhc2dcb LOC571282 ccr9b itgb7 si:dkey-1h24.6 ccl25a | dre:799354 dre:799354 dre:30762 dre:368878 dre:100006078 dre:30763 dre:571282 dre:100006078 dre:797491 dre:100151365 dre:100333914 |
| dre01200 | Carbon metabolism | 32/900 | 173/8379 | 0.00142360053742801 | 0.0562322212284064 | 335715 560753\_1 560753 116517 568001\_2 445233 322670\_1 550579 368262 560827\_1 406762\_1 403070\_1 437019\_1 541329 570579\_1 407673 100006589\_1 541329\_1 550579\_1 403070 561416\_1 792824\_1 317746 406339\_1 561416 406688 393427 406702 563642 321224 751668 568001\_3 | mdh1ab tpi1b tpi1b adh5 pfkmb me2 aco2 idh3g dlst prps1b pgd rgn pcca gcsha g6pd rpia idh1 gcsha idh3g rgn pfkpb pgp suclg2 hk2 pfkpb got2a zgc:63568 pdha1a sdsl hkdc1 gck pfkmb | dre:335715 dre:560753 dre:560753 dre:116517 dre:568001 dre:445233 dre:322670 dre:550579 dre:368262 dre:560827 dre:406762 dre:403070 dre:437019 dre:541329 dre:570579 dre:407673 dre:100006589 dre:541329 dre:550579 dre:403070 dre:561416 dre:792824 dre:317746 dre:406339 dre:561416 dre:406688 dre:393427 dre:406702 dre:563642 dre:321224 dre:751668 dre:568001 |
| dre00010 | Glycolysis / Gluconeogenesis | 20/900 | 103/8379 | 0.00604291125401808 | 0.171739526656222 | 560753\_1 560753 100005587\_1 116517 100536795 568001\_2 406473 368239 561416\_1 406339\_1 561416 393427 406702 445326\_1 445326 321224 436646 751668 100006238\_1 568001\_3 | tpi1b tpi1b aldh9a1a.1 adh5 wu:fb14d09 pfkmb pck2 aldh2.2 pfkpb hk2 pfkpb zgc:63568 pdha1a akr1a1a akr1a1a hkdc1 galm gck aldh9a1a.2 pfkmb | dre:560753 dre:560753 dre:100005587 dre:116517 dre:100536795 dre:568001 dre:406473 dre:368239 dre:561416 dre:406339 dre:561416 dre:393427 dre:406702 dre:445326 dre:445326 dre:321224 dre:436646 dre:751668 dre:100006238 dre:568001 |
| dre00591 | Linoleic acid metabolism | 9/900 | 33/8379 | 0.00652175417681855 | 0.171739526656222 | 406370\_1 406370 393592 553969 797309 560649 399485 767688 100148818 | pla2g6 pla2g6 cyp2p9 cyp3a65 cyp2j20 si:ch211-214p16.3 cyp2p10 zgc:154040 si:ch73-55i23.1 | dre:406370 dre:406370 dre:393592 dre:553969 dre:797309 dre:560649 dre:399485 dre:767688 dre:100148818 |
| dre03018 | RNA degradation | 20/900 | 108/8379 | 0.0102806571313345 | 0.199469503398001 | 373085\_1 448949\_1 568001\_2 394064\_1 553665 324574 448949 553665\_1 566017 100538016 492479\_1 565000 565659 561416\_1 317641\_1 566017\_1 565659\_1 561416 321035\_1 568001\_3 | hspa9 cnot1 pfkmb exosc10 cnot6l wu:fc33b09 cnot1 cnot6l lsm8 si:dkey-79d12.5 btg3 exosc3 edc4 pfkpb zcchc7 lsm8 edc4 pfkpb pabpc4 pfkmb | dre:373085 dre:448949 dre:568001 dre:394064 dre:553665 dre:324574 dre:448949 dre:553665 dre:566017 dre:100538016 dre:492479 dre:324574 dre:565659 dre:561416 dre:317641 dre:566017 dre:565659 dre:561416 dre:321035 dre:568001 |
| dre00380 | Tryptophan metabolism | 14/900 | 67/8379 | 0.0105491683239868 | 0.199469503398001 | 100005587\_1 100536795 103911233 368262 447801\_1 393860\_1 447801 368239 571201 393860 571967 555401 103911241 100006238\_1 | aldh9a1a.1 wu:fb14d09 LOC103911233 dlst aldh8a1 gcdha aldh8a1 aldh2.2 si:ch73-97h19.2 gcdha ido1 aoc1 LOC103911241 aldh9a1a.2 | dre:100005587 dre:100536795 dre:103911233 dre:368262 dre:447801 dre:393860 dre:447801 dre:368239 dre:571201 dre:393860 dre:571967 dre:555401 dre:571201 dre:100006238 |
| dre01230 | Biosynthesis of amino acids | 20/900 | 109/8379 | 0.0113621869024178 | 0.199469503398001 | 334195\_1 560753\_1 560753 568001\_2 322670\_1 550579 560827\_1 550524\_1 407673 100006589\_1 550579\_1 393799 561416\_1 445818 561416 406688 563642 334195 678634 568001\_3 | mat1a tpi1b tpi1b pfkmb aco2 idh3g prps1b cbsa rpia idh1 idh3g pycr1a pfkpb cthl pfkpb got2a sdsl mat1a mat2al pfkmb | dre:334195 dre:560753 dre:560753 dre:568001 dre:322670 dre:550579 dre:560827 dre:550524 dre:407673 dre:100006589 dre:550579 dre:393799 dre:561416 dre:445818 dre:561416 dre:406688 dre:563642 dre:334195 dre:678634 dre:568001 |
| dre00260 | Glycine, serine and threonine metabolism | 12/900 | 56/8379 | 0.0141677605110368 | 0.223850616074382 | 394103\_1 541429\_1 541429 550524\_1 541329 541329\_1 445818 394103 405800 619259 563642 64607\_1 | sardh aoc2 aoc2 cbsa gcsha gcsha cthl sardh dao.2 dao.1 sdsl alas2 | dre:394103 dre:541429 dre:541429 dre:550524 dre:541329 dre:541329 dre:445818 dre:394103 dre:405800 dre:619259 dre:563642 dre:64607 |
| dre00900 | Terpenoid backbone biosynthesis | 7/900 | 26/8379 | 0.01701895014937 | 0.229348476250723 | 334380\_1 559054\_1 334380 559054 557720\_1 100003383\_1 100003383 | zmpste24 hmgcra zmpste24 hmgcra rce1a pmvk pmvk | dre:334380 dre:559054 dre:334380 dre:559054 dre:557720 dre:100003383 dre:100003383 |
| dre04150 | mTOR signaling pathway | 38/900 | 251/8379 | 0.0178197305518563 | 0.229348476250723 | 324140\_1 324140 796322 336619 436718\_1 100333504\_1 436718 79380 359840\_1 100000252\_1 573492 792689\_1 799099\_1 79380\_1 359840 100000252\_3 100006675 553214\_1 245702\_1 563708 556087\_1 555584 100330617\_1 58072 30369\_1 555584\_1 791993 790938 30127\_1 100000252 359839 245702 797250 100009657\_1 556087 58072\_1 497384\_1 573492\_1 | sgk1 sgk1 slc3a2a eif4bb rhocb lamtor5 rhocb eif4ea atp6v1b2 pik3r3b rragca mlst8 lpin1 eif4ea atp6v1b2 pik3r3b prkcba eif4ba igf1rb skp2 wnt2bb prr5a tsc1b fzd8b fzd7a prr5a wnt4b zgc:158423 wnt2 pik3r3b atp6v1ba igf1rb slc7a5 rps6ka3b wnt2bb fzd8b prkca rragca | dre:324140 dre:324140 dre:796322 dre:336619 dre:436718 dre:100333504 dre:436718 dre:79380 dre:359840 dre:100000252 dre:573492 dre:792689 dre:799099 dre:79380 dre:359840 dre:100000252 dre:100006675 dre:553214 dre:245702 dre:563708 dre:556087 dre:555584 dre:100330617 dre:58072 dre:30369 dre:555584 dre:791993 dre:790938 dre:30127 dre:100000252 dre:359839 dre:245702 dre:797250 dre:100009657 dre:556087 dre:58072 dre:497384 dre:573492 |
| dre00030 | Pentose phosphate pathway | 10/900 | 45/8379 | 0.0188704442484772 | 0.229348476250723 | 568001\_2 560827\_1 406762\_1 403070\_1 570579\_1 407673 403070 561416\_1 561416 568001\_3 | pfkmb prps1b pgd rgn g6pd rpia rgn pfkpb pfkpb pfkmb | dre:568001 dre:560827 dre:406762 dre:403070 dre:570579 dre:407673 dre:403070 dre:561416 dre:561416 dre:568001 |
| dre03440 | Homologous recombination | 10/900 | 46/8379 | 0.0218680551075194 | 0.246445582607916 | 558923\_1 558923 406487 794038\_1 554171 404608 794038 406721 554171\_1 450081 | palb2 palb2 rad51 brip1 rad52 rad51d brip1 rad51b rad52 rad51c | dre:558923 dre:558923 dre:406487 dre:794038 dre:554171 dre:404608 dre:794038 dre:406721 dre:554171 dre:450081 |
| dre00360 | Phenylalanine metabolism | 6/900 | 22/8379 | 0.0249565146944725 | 0.246445582607916 | 541429\_1 541429 561410\_1 445287 394142 406688 | aoc2 aoc2 tat hpdb hpda got2a | dre:541429 dre:541429 dre:561410 dre:445287 dre:394142 dre:406688 |
| dre00450 | Selenocompound metabolism | 6/900 | 22/8379 | 0.0249565146944725 | 0.246445582607916 | 103911233 777719 571201 101884223 445818 103911241 | LOC103911233 papss2a si:ch73-97h19.2 LOC101884223 cthl LOC103911241 | dre:103911233 dre:777719 dre:571201 dre:101884223 dre:445818 dre:571201 |
| dre00620 | Pyruvate metabolism | 13/900 | 68/8379 | 0.0271513821532152 | 0.252348140012236 | 335715 100005587\_1 116517 100536795 445233 406473 368239 393427 559403\_1 406702 445326\_1 445326 100006238\_1 | mdh1ab aldh9a1a.1 adh5 wu:fb14d09 me2 pck2 aldh2.2 zgc:63568 acaca pdha1a akr1a1a akr1a1a aldh9a1a.2 | dre:335715 dre:100005587 dre:116517 dre:100536795 dre:445233 dre:406473 dre:368239 dre:393427 dre:559403 dre:406702 dre:445326 dre:445326 dre:100006238 |
| dre00052 | Galactose metabolism | 9/900 | 43/8379 | 0.0361638086123474 | 0.310507037350867 | 568001\_2 561416\_1 406339\_1 450083 561416 321224 436646 751668 568001\_3 | pfkmb pfkpb hk2 gla pfkpb hkdc1 galm gck pfkmb | dre:568001 dre:561416 dre:406339 dre:450083 dre:561416 dre:321224 dre:436646 dre:751668 dre:568001 |
| dre00350 | Tyrosine metabolism | 10/900 | 50/8379 | 0.0373394538586486 | 0.310507037350867 | 116517 541429\_1 541429 561410\_1 445287 394142 406688 393427 569363 30505 | adh5 aoc2 aoc2 tat hpdb hpda got2a zgc:63568 tpo dbh | dre:116517 dre:541429 dre:541429 dre:561410 dre:445287 dre:394142 dre:406688 dre:393427 dre:569363 dre:30505 |
| dre00500 | Starch and sucrose metabolism | 9/900 | 44/8379 | 0.0413010515654314 | 0.326278307366908 | 393444 406539 445049 100333665 406339\_1 100333665\_1 321224 561803 751668 | pygmb amy2a zgc:92137 gbe1a hk2 gbe1a hkdc1 gyg2 gck | dre:393444 dre:406539 dre:445049 dre:100333665 dre:406339 dre:100333665 dre:321224 dre:561803 dre:751668 |
| dre00053 | Ascorbate and aldarate metabolism | 12/900 | 66/8379 | 0.0464965081847068 | 0.338448309509641 | 100005587\_1 100536795 403070\_1 368239 403070 406731 100415796 570913\_1 445326\_1 445326 767756 100006238\_1 | aldh9a1a.1 wu:fb14d09 rgn aldh2.2 rgn ugt1ab ugt5b5 ugt5g1 akr1a1a akr1a1a ugt5a2 aldh9a1a.2 | dre:100005587 dre:100536795 dre:403070 dre:368239 dre:403070 dre:406731 dre:678597 dre:570913 dre:445326 dre:445326 dre:767756 dre:100006238 |
| dre04070 | Phosphatidylinositol signaling system | 24/900 | 157/8379 | 0.047125713982355 | 0.338448309509641 | 724010 100002015\_1 336121 724010\_1 368717 368717\_1 100000252\_1 337236\_1 337236\_2 559938\_1 100000252\_3 100006675 337236 566642\_1 566642 559938 571356 678558\_1 571356\_1 571356\_2 559198\_1 100000252 559198 497384\_1 | dgkaa cds1 calm2a dgkaa itpka itpka pik3r3b synj1 synj1 ppip5k1b pik3r3b prkcba synj1 tmem55a tmem55a ppip5k1b pik3c2a mtmr7a pik3c2a pik3c2a mtmr7b pik3r3b mtmr7b prkca | dre:724010 dre:100002015 dre:192322 dre:724010 dre:368717 dre:368717 dre:100000252 dre:337236 dre:337236 dre:559938 dre:100000252 dre:100006675 dre:337236 dre:566642 dre:566642 dre:559938 dre:571356 dre:678558 dre:571356 dre:571356 dre:559198 dre:100000252 dre:559198 dre:497384 |
| dre00592 | alpha-Linolenic acid metabolism | 6/900 | 26/8379 | 0.0533315479958038 | 0.366364547101609 | 406370\_1 406370 140615 560649 767688 100148818 | pla2g6 pla2g6 fads2 si:ch211-214p16.3 zgc:154040 si:ch73-55i23.1 | dre:406370 dre:406370 dre:140615 dre:560649 dre:767688 dre:100148818 |
| dre00051 | Fructose and mannose metabolism | 10/900 | 54/8379 | 0.0590947458583079 | 0.376574464544938 | 560753\_1 560753 568001\_2 561416\_1 406339\_1 436909 561416 100007686 321224 568001\_3 | tpi1b tpi1b pfkmb pfkpb hk2 khk pfkpb LOC100007686 hkdc1 pfkmb | dre:560753 dre:560753 dre:568001 dre:561416 dre:406339 dre:436909 dre:561416 dre:100007686 dre:321224 dre:568001 |
| dre00020 | Citrate cycle (TCA cycle) | 9/900 | 47/8379 | 0.059584567174832 | 0.376574464544938 | 335715 322670\_1 550579 406473 368262 100006589\_1 550579\_1 317746 406702 | mdh1ab aco2 idh3g pck2 dlst idh1 idh3g suclg2 pdha1a | dre:335715 dre:322670 dre:550579 dre:406473 dre:368262 dre:100006589 dre:550579 dre:317746 dre:406702 |
| dre03460 | Fanconi anemia pathway | 11/900 | 63/8379 | 0.0703734012861212 | 0.424537231617418 | 558923\_1 100538153 558923 406487 794038\_1 402936 100538153\_1 794038 402936\_1 450081 570043\_1 | palb2 slx4 palb2 rad51 brip1 fancg slx4 brip1 fancg rad51c rev1 | dre:558923 dre:100538153 dre:558923 dre:406487 dre:794038 dre:402936 dre:100538153 dre:794038 dre:402936 dre:450081 dre:570043 |
| dre04130 | SNARE interactions in vesicular transport | 10/900 | 56/8379 | 0.0725475016055081 | 0.424537231617418 | 567385\_1 449923 436605 449923\_1 393836 571872 393294 325484 393236 565822\_1 | vti1a si:ch73-234b20.5 stx5a si:ch73-234b20.5 stx11b.1 zgc:165520 use1 vamp5 sybl1 bnip1a | dre:567385 dre:449923 dre:436605 dre:449923 dre:393836 dre:571872 dre:393294 dre:325484 dre:393236 dre:565822 |
| dre04810 | Regulation of actin cytoskeleton | 52/900 | 404/8379 | 0.0931454267135431 | 0.521373206798121 | 57934\_1 57934 286739\_1 799354 30429\_2 793158 570868\_1 436718\_1 286739 566764 436718 566764\_1 100000252\_1 557935 793158\_1 386856\_1 100000252\_3 799354\_1 100331581 100002872\_2 100005049 novel.946 677750 100331581\_1 569566 571431 560564\_2 556226\_1 796490\_2 100536705\_2 494456 100005469\_1 553493 570832 323285\_1 378480 553493\_1 393611 445305\_1 30429 58075 100000252 767646\_1 794365 103909601 797491\_1 564565 798694\_1 570832\_1 564266 792699 101882911 | actb1 actb1 msna cxcl12b mylpfa cdc42l itga1 rhocb msna scin rhocb scin pik3r3b zmp:0000001082 cdc42l pdgfra pik3r3b cxcl12b si:dkey-44g17.6 cyfip2 fgf1b - myl2b si:dkey-44g17.6 LOC569566 LOC571431 pdgfc cdc42l2 pdgfbb itgae.2 fgf23 fn1a arhgef7a c7b scinla pdgfaa arhgef7a c6 arpc4l mylpfa fgd pik3r3b abi2a si:dkey-163m14.7 LOC103909601 itgb7 arhgap35 LOC798694 c7b itgb3a rac1l LOC101882911 | dre:57934 dre:57934 dre:286739 dre:799354 dre:30429 dre:793158 dre:570868 dre:436718 dre:286739 dre:566764 dre:436718 dre:566764 dre:100000252 dre:557935 dre:793158 dre:386856 dre:100000252 dre:799354 dre:100331581 dre:100002872 dre:100005049 dre:386856 dre:677750 dre:100331581 dre:569566 dre:571431 dre:560564 dre:556226 dre:796490 dre:100536705 dre:494456 dre:100005469 dre:553493 dre:570832 dre:323285 dre:103909601 dre:553493 dre:393611 dre:445305 dre:30429 dre:58075 dre:100000252 dre:767646 dre:794365 dre:103909601 dre:797491 dre:564565 dre:798694 dre:570832 dre:564266 dre:792699 dre:321070 |
| dre03430 | Mismatch repair | 6/900 | 30/8379 | 0.0956950822604146 | 0.521373206798121 | 556995\_1 568323 556995\_2 565400 568323\_1 565400\_1 | lig1 mlh3 lig1 msh3 mlh3 msh3 | dre:556995 dre:568323 dre:556995 dre:565400 dre:568323 dre:565400 |
| dre01232 | Nucleotide metabolism | 19/900 | 131/8379 | 0.106892823876134 | 0.548036305929737 | 100330864 492517\_1 334431\_1 492517 100005763\_1 550560\_1 100005763 437016\_1 103910413\_1 393744\_1 553639\_1 103910413 402953\_1 333997 558466\_1 100005067\_1 393744\_2 447905 333997\_1 | LOC100330864 zgc:103586 adsl zgc:103586 uck2a entpd6 uck2a tk2 tymp dctpp1 gmpr tymp pnp6 ampd3b uckl1b dguok dctpp1 entpd2a.1 ampd3b | dre:100330864 dre:492517 dre:334431 dre:492517 dre:100005763 dre:550560 dre:100005763 dre:437016 dre:103910413 dre:393744 dre:553639 dre:103910413 dre:402953 dre:333997 dre:558466 dre:100005067 dre:393744 dre:447905 dre:333997 |
| dre00100 | Steroid biosynthesis | 5/900 | 24/8379 | 0.1075261106571 | 0.548036305929737 | 322481 100004700 565117 406662\_2 494054\_1 | cel.1 cyp24a1 cel.2 msmo1 tm7sf2 | dre:322481 dre:100004700 dre:565117 dre:406662 dre:494054 |
| dre04210 | Apoptosis | 31/900 | 232/8379 | 0.116919615910524 | 0.57729060355821 | 57934\_1 57934 140621 373107\_1 431763\_1 791531\_1 431763 100000252\_1 556410\_1 100000252\_3 552927\_1 394198 570238 567021 569298 564295\_2 140621\_1 101886621\_1 768248 58100\_1 561021 559564 552927 58133 431777 791531 100000252 561021\_1 100135062\_2 246726 558237\_1 | actb1 actb1 casp3a birc2 gadd45ab casp6l1 gadd45ab pik3r3b atf4b pik3r3b casp6 fosab fosaa ern2 ctsbb traf2a casp3a LOC101886621 fas badb si:dkey-103e21.5 eif2ak3 casp6 ngfb tuba7l casp6l1 pik3r3b si:dkey-103e21.5 csf2rb birc5b dab2ipb | dre:57934 dre:57934 dre:140621 dre:373107 dre:431763 dre:791531 dre:431763 dre:100000252 dre:556410 dre:100000252 dre:552927 dre:394198 dre:570238 dre:567021 dre:569298 dre:564295 dre:140621 dre:101886621 dre:768248 dre:58100 dre:561021 dre:559564 dre:552927 dre:58133 dre:431777 dre:791531 dre:100000252 dre:561021 dre:100135062 dre:246726 dre:558237 |
| dre03015 | mRNA surveillance pathway | 17/900 | 118/8379 | 0.127721778780983 | 0.586454918405057 | 386630 324267 386630\_1 324267\_1 393693\_1 100005893 393693 100005893\_1 492341\_1 394058\_1 563172 100002680\_2 562282\_1 321035\_1 559392\_1 562282 559392 | tardbpl hbs1l tardbpl hbs1l sap18 magoh sap18 magoh pcf11 fus etf1a msi2a ppp2r5b pabpc4 ppp2r2cb ppp2r5b ppp2r2cb | dre:386630 dre:324267 dre:386630 dre:324267 dre:393693 dre:100005893 dre:393693 dre:100005893 dre:492341 dre:394058 dre:563172 dre:100002680 dre:562282 dre:321035 dre:559392 dre:562282 dre:559392 |
| dre00470 | D-Amino acid metabolism | 3/900 | 12/8379 | 0.130369361082123 | 0.586454918405057 | 560387 405800 619259 | c17h14orf159 dao.2 dao.1 | dre:560387 dre:405800 dre:619259 |
| dre00920 | Sulfur metabolism | 3/900 | 12/8379 | 0.130369361082123 | 0.586454918405057 | 777719 393542\_1 566675 | papss2a selenbp1 suox | dre:777719 dre:393542 dre:566675 |
| dre02010 | ABC transporters | 10/900 | 63/8379 | 0.133622639636595 | 0.586454918405057 | 368620\_1 406803 553281 570453\_1 406803\_1 406803\_2 571189 570148\_1 735310 556826\_1 | abcc4 abcd3a abcc8 abcg2b abcd3a abcd3a abcb11b abcb9 abcg2d si:dkey-57h18.2 | dre:368620 dre:406803 dre:553281 dre:570453 dre:406803 dre:406803 dre:571189 dre:570148 dre:735310 dre:556826 |
| dre04370 | VEGF signaling pathway | 15/900 | 104/8379 | 0.14482259284247 | 0.618431612678654 | 793158 565241\_2 559020 562552 100000252\_1 793158\_1 100000252\_3 100006675 556226\_1 58100\_1 565241 100148818 100000252 497384\_1 792699 | cdc42l ppp3ccb ptgs2b zgc:171775 pik3r3b cdc42l pik3r3b prkcba cdc42l2 badb ppp3ccb si:ch73-55i23.1 pik3r3b prkca rac1l | dre:793158 dre:565241 dre:559020 dre:562552 dre:100000252 dre:793158 dre:100000252 dre:100006675 dre:556226 dre:58100 dre:565241 dre:100148818 dre:100000252 dre:497384 dre:792699 |
| dre00561 | Glycerolipid metabolism | 13/900 | 89/8379 | 0.155317799536595 | 0.631188182091813 | 724010 100005587\_1 100536795 724010\_1 322481 368239 799099\_1 565117 450083 445326\_1 572841 445326 100006238\_1 | dgkaa aldh9a1a.1 wu:fb14d09 dgkaa cel.1 aldh2.2 lpin1 cel.2 gla akr1a1a mboat1 akr1a1a aldh9a1a.2 | dre:724010 dre:100005587 dre:100536795 dre:724010 dre:322481 dre:368239 dre:799099 dre:565117 dre:450083 dre:445326 dre:572841 dre:445326 dre:100006238 |
| dre00330 | Arginine and proline metabolism | 12/900 | 81/8379 | 0.155799614566966 | 0.631188182091813 | 100005587\_1 100536795 321892 321892\_1 368239 393799 405800 406688 555401 619259 327506\_1 100006238\_1 | aldh9a1a.1 wu:fb14d09 ckmt1 ckmt1 aldh2.2 pycr1a dao.2 got2a aoc1 dao.1 ckmt2a aldh9a1a.2 | dre:100005587 dre:100536795 dre:321892 dre:321892 dre:368239 dre:393799 dre:405800 dre:406688 dre:555401 dre:619259 dre:327506 dre:100006238 |
| dre00603 | Glycosphingolipid biosynthesis - globo and isoglobo series | 4/900 | 20/8379 | 0.160507415021088 | 0.634004289333297 | 447881 450083 100093713 100005375 | gbgt1l3 gla zgc:165519 si:ch211-135f11.4 | dre:447881 dre:450083 dre:100093713 dre:100005375 |
| dre03410 | Base excision repair | 9/900 | 58/8379 | 0.165168631963745 | 0.636503508543213 | 321622 110438871 393774\_2 393774 556995\_1 556995\_2 553405\_1 558198\_1 796446\_1 | hmgb1a LOC110438871 pole3 pole3 lig1 lig1 pole si:ch211-141o9.10 adprhl2 | dre:110438871 dre:110438871 dre:393774 dre:393774 dre:556995 dre:556995 dre:553405 dre:558198 dre:796446 |
| dre00512 | Mucin type O-glycan biosynthesis | 7/900 | 43/8379 | 0.173106334047488 | 0.641713064194978 | 324510\_1 324510 557675 557675\_1 555344 337131 568500 | gcnt4a gcnt4a c1galt1a c1galt1a si:dkey-202e17.1 c1galt1b galnt8a.2 | dre:324510 dre:324510 dre:557675 dre:557675 dre:555344 dre:337131 dre:568500 |
| dre00983 | Drug metabolism - other enzymes | 17/900 | 124/8379 | 0.17464342886319 | 0.641713064194978 | 100330864 492517\_1 492517 406731 100005763\_1 100005763 437016\_1 794381 103910413\_1 100415796 393493 570913\_1 406703 103910413 558466\_1 431762\_1 767756 | LOC100330864 zgc:103586 zgc:103586 ugt1ab uck2a uck2a tk2 si:dkey-78a14.4 tymp ugt5b5 gstt2 ugt5g1 gsta.1 tymp uckl1b mgst1.2 ugt5a2 | dre:100330864 dre:492517 dre:492517 dre:406731 dre:100005763 dre:100005763 dre:437016 dre:794381 dre:103910413 dre:678597 dre:393493 dre:570913 dre:406703 dre:103910413 dre:558466 dre:431762 dre:767756 |
| dre00130 | Ubiquinone and other terpenoid-quinone biosynthesis | 3/900 | 14/8379 | 0.184323379510121 | 0.656099152710654 | 561410\_1 445287 394142 | tat hpdb hpda | dre:561410 dre:445287 dre:394142 |
| dre00240 | Pyrimidine metabolism | 12/900 | 84/8379 | 0.186863682734047 | 0.656099152710654 | 100330864 492517\_1 492517 100005763\_1 550560\_1 100005763 437016\_1 103910413\_1 393744\_1 103910413 558466\_1 393744\_2 | LOC100330864 zgc:103586 zgc:103586 uck2a entpd6 uck2a tk2 tymp dctpp1 tymp uckl1b dctpp1 | dre:100330864 dre:492517 dre:492517 dre:100005763 dre:550560 dre:100005763 dre:437016 dre:103910413 dre:393744 dre:103910413 dre:558466 dre:393744 |
| dre00310 | Lysine degradation | 14/900 | 102/8379 | 0.202419322472428 | 0.692389298538213 | 100005587\_1 100536795 368262 393860\_1 368239 393860 556077\_1 556535\_1 100003615 100003615\_1 572649 402830 100006238\_1 556535 | aldh9a1a.1 wu:fb14d09 dlst gcdha aldh2.2 gcdha plod3 setd1a prdm2a prdm2a bbox1 ehmt1b aldh9a1a.2 setd1a | dre:100005587 dre:100536795 dre:368262 dre:393860 dre:368239 dre:393860 dre:556077 dre:556535 dre:100003615 dre:100003615 dre:572649 dre:402830 dre:100006238 dre:556535 |
| dre05132 | Salmonella infection | 49/900 | 406/8379 | 0.208774694368565 | 0.692389298538213 | 57934\_1 386590\_1 386590 57934 562529\_1 30429\_2 793158 140621 436718\_1 373107\_1 100004870\_1 562372\_1 558917\_1 562552 436718 563023\_1 793158\_1 406811\_1 394198 570238 798985 563023 436734\_1 798985\_1 100002872\_2 101882034 677750 566185 562372 393653\_1 564295\_2 140621\_1 556226\_1 571356 571356\_1 561021 571356\_2 novel.848 110437957 548603\_1 431777 100002946 445305\_1 30429 561021\_1 403131 792699 799806\_2 101882911 | actb1 hsp90b1 hsp90b1 actb1 flna mylpfa cdc42l casp3a rhocb birc2 exoc2 snx18a snx9b zgc:171775 rhocb flnb cdc42l zgc:55461 fosab fosaa vps16 flnb txn vps16 cyfip2 LOC101882034 myl2b caspbl snx18a rhogd traf2a casp3a cdc42l2 pik3c2a pik3c2a si:dkey-103e21.5 pik3c2a - LOC110437957 snx18b tuba7l cxcl8a arpc4l mylpfa si:dkey-103e21.5 tlr4ba rac1l anxa2b LOC101882911 | dre:57934 dre:386590 dre:386590 dre:57934 dre:562529 dre:30429 dre:793158 dre:140621 dre:436718 dre:373107 dre:100004870 dre:562372 dre:558917 dre:562552 dre:436718 dre:563023 dre:793158 dre:336681 dre:394198 dre:570238 dre:798985 dre:563023 dre:436734 dre:798985 dre:100002872 dre:406297 dre:677750 dre:566185 dre:562372 dre:393653 dre:564295 dre:140621 dre:556226 dre:571356 dre:571356 dre:561021 dre:571356 dre:393993 dre:373874 dre:548603 dre:431777 dre:100002946 dre:445305 dre:30429 dre:561021 dre:403131 dre:792699 dre:799806 dre:321070 |
| dre00340 | Histidine metabolism | 5/900 | 30/8379 | 0.214728326761851 | 0.692389298538213 | 100005587\_1 100536795 368239 555401 100006238\_1 | aldh9a1a.1 wu:fb14d09 aldh2.2 aoc1 aldh9a1a.2 | dre:100005587 dre:100536795 dre:368239 dre:555401 dre:100006238 |
| dre01210 | 2-Oxocarboxylic acid metabolism | 5/900 | 30/8379 | 0.214728326761851 | 0.692389298538213 | 322670\_1 550579 100006589\_1 550579\_1 406688 | aco2 idh3g idh1 idh3g got2a | dre:322670 dre:550579 dre:100006589 dre:550579 dre:406688 |
| dre04120 | Ubiquitin mediated proteolysis | 25/900 | 199/8379 | 0.230252891652233 | 0.717534502525822 | 550134 192341 564149 550134\_1 373107\_1 692325 692325\_1 564149\_1 novel.905 323951 323883 327215\_2 100136859 323951\_2 100001302 447935\_1 556277 563708 564866\_1 559439\_1 393223\_1 393445\_1 30100 559981 100151173\_1 | ubb tceb2 pias2 ubb birc2 flj11011l flj11011l pias2 - klhl13 cul1a cul5a fbxw8 klhl13 uba7 tceb1a cblb skp2 trip12 huwe1 fzr1a ube3c ube2kb si:ch73-190m4.1 si:rp71-68n21.9 | dre:550134 dre:192341 dre:564149 dre:550134 dre:373107 dre:692325 dre:692325 dre:564149 dre:777766 dre:323951 dre:323883 dre:327215 dre:100136859 dre:323951 dre:100001302 dre:447935 dre:556277 dre:563708 dre:564866 dre:559439 dre:393223 dre:393445 dre:30100 dre:559981 dre:100151173 |
| dre01240 | Biosynthesis of cofactors | 29/900 | 234/8379 | 0.231609238157069 | 0.717534502525822 | 334195\_1 100536795 334431\_1 557552 403070\_1 368239 403070 445287 406731 394142 571967 100004977\_2 445083 393580 100415796 100007686 570913\_1 406789 407663 445326\_1 64607\_1 110438097 100330410 574009 450084\_1 334195 445326 767756 678634 | mat1a wu:fb14d09 adsl zgc:153031 rgn aldh2.2 rgn hpdb ugt1ab hpda ido1 mthfd2 dhrs3a bco1l ugt5b5 LOC100007686 ugt5g1 pank1a si:ch211-107o10.3 akr1a1a alas2 LOC110438097 alp3 alpi.2 gss mat1a akr1a1a ugt5a2 mat2al | dre:334195 dre:100536795 dre:334431 dre:557552 dre:403070 dre:368239 dre:403070 dre:445287 dre:406731 dre:394142 dre:571967 dre:100004977 dre:445083 dre:393580 dre:678597 dre:100007686 dre:570913 dre:406789 dre:407663 dre:445326 dre:64607 dre:572070 dre:100330410 dre:574009 dre:450084 dre:334195 dre:445326 dre:767756 dre:678634 |
| dre03030 | DNA replication | 7/900 | 48/8379 | 0.252903691475454 | 0.764009554207146 | 393241\_1 393774\_2 393774 556995\_1 393241\_2 556995\_2 553405\_1 | rnaseh2b pole3 pole3 lig1 rnaseh2b lig1 pole | dre:393241 dre:393774 dre:393774 dre:556995 dre:393241 dre:556995 dre:553405 |
| dre05168 | Herpes simplex virus 1 infection | 27/900 | 220/8379 | 0.257997538640414 | 0.764009554207146 | 140621 373107\_1 100149189 100000252\_1 565338\_1 100000252\_3 30400 564295\_2 30762 140621\_1 406288\_1 58113 368878 100330617\_1 768248 58100\_1 561021 30763 559564 368481 30163 791704 100000252 571282 561021\_1 564266 556826\_1 | casp3a birc2 si:ch73-237c6.1 pik3r3b eif2ak1 pik3r3b b2m traf2a mhc2dab casp3a srsf1a cd74a si:busm1-266f07.2 tsc1b fas badb si:dkey-103e21.5 mhc2dcb eif2ak3 stat1b tapbp.1 zgc:153759 pik3r3b LOC571282 si:dkey-103e21.5 itgb3a si:dkey-57h18.2 | dre:140621 dre:373107 dre:100149189 dre:100000252 dre:565338 dre:100000252 dre:30400 dre:564295 dre:30762 dre:140621 dre:406288 dre:58113 dre:368878 dre:100330617 dre:768248 dre:58100 dre:561021 dre:30763 dre:559564 dre:368481 dre:30163 dre:791704 dre:100000252 dre:571282 dre:561021 dre:564266 dre:556826 |
| dre04137 | Mitophagy - animal | 15/900 | 116/8379 | 0.261117189412569 | 0.764009554207146 | 550134 100000242 550134\_1 novel.905 556410\_1 555713\_1 555713 326974\_1 564996 492676 336159\_1 559564 561933\_2 561933 561933\_1 | ubb zgc:92606 ubb - atf4b nbr1b nbr1b gabarapa tfe3b tbc1d15 optn eif2ak3 rhot1b rhot1b rhot1b | dre:550134 dre:100000242 dre:550134 dre:777766 dre:556410 dre:555713 dre:555713 dre:326974 dre:564996 dre:492676 dre:336159 dre:559564 dre:561933 dre:561933 dre:561933 |
| dre00410 | beta-Alanine metabolism | 6/900 | 41/8379 | 0.27435198650361 | 0.7838325341102 | 100005587\_1 541429\_1 100536795 541429 368239 100006238\_1 | aldh9a1a.1 aoc2 wu:fb14d09 aoc2 aldh2.2 aldh9a1a.2 | dre:100005587 dre:541429 dre:100536795 dre:541429 dre:368239 dre:100006238 |
| dre03060 | Protein export | 4/900 | 25/8379 | 0.278204784388519 | 0.7838325341102 | 445299 436794\_2 436794 445299\_1 | immp2l sec11a sec11a immp2l | dre:445299 dre:436794 dre:436794 dre:445299 |
| dre04115 | p53 signaling pathway | 13/900 | 101/8379 | 0.286620818821334 | 0.7838325341102 | 100330864 140621 431763\_1 431763 171473 436699 436699\_1 140621\_1 768248 561021 796473\_1 100320152 561021\_1 | LOC100330864 casp3a gadd45ab gadd45ab ccng1 zgc:92658 zgc:92658 casp3a fas si:dkey-103e21.5 tp53i3 steap3 si:dkey-103e21.5 | dre:100330864 dre:140621 dre:431763 dre:431763 dre:171473 dre:436699 dre:436699 dre:140621 dre:768248 dre:561021 dre:796473 dre:100320152 dre:561021 |
| dre00565 | Ether lipid metabolism | 9/900 | 67/8379 | 0.289930481906719 | 0.7838325341102 | 406370\_1 406370 492735 565743\_1 560225 560649 767688 100148818 567170\_1 | pla2g6 pla2g6 si:ch211-212o1.2 pld2 cept1a si:ch211-214p16.3 zgc:154040 si:ch73-55i23.1 pafah1b2 | dre:406370 dre:406370 dre:492735 dre:565743 dre:560225 dre:560649 dre:767688 dre:100148818 dre:567170 |
| dre00400 | Phenylalanine, tyrosine and tryptophan biosynthesis | 2/900 | 10/8379 | 0.292696958939885 | 0.7838325341102 | 561410\_1 406688 | tat got2a | dre:561410 dre:406688 |
| dre00740 | Riboflavin metabolism | 2/900 | 11/8379 | 0.334215354235107 | 0.880100432819115 | 436725\_1 436725 | acp5b acp5b | dre:436725 dre:436725 |
| dre00562 | Inositol phosphate metabolism | 15/900 | 123/8379 | 0.34101984733714 | 0.883297309496199 | 560753\_1 560753 368717 368717\_1 337236\_1 337236\_2 337236 100006223 571356 678558\_1 566479\_1 571356\_1 571356\_2 559198\_1 559198 | tpi1b tpi1b itpka itpka synj1 synj1 synj1 si:dkey-266f7.9 pik3c2a mtmr7a plch1 pik3c2a pik3c2a mtmr7b mtmr7b | dre:560753 dre:560753 dre:368717 dre:368717 dre:337236 dre:337236 dre:337236 dre:100006223 dre:571356 dre:678558 dre:566479 dre:571356 dre:571356 dre:559198 dre:559198 |
| dre03320 | PPAR signaling pathway | 13/900 | 106/8379 | 0.34942942625107 | 0.888488676503874 | 550134 550134\_1 406473 30355 723999 novel.905 334529 30355\_1 553512 140615 794841\_1 795106 550432 | ubb ubb pck2 apoa1a zgc:136333 - aqp7 apoa1a slc27a1b fads2 plin2 cyp27a1.2 slc27a4 | dre:550134 dre:550134 dre:406473 dre:30355 dre:795106 dre:777766 dre:334529 dre:30355 dre:553512 dre:140615 dre:794841 dre:795106 dre:550432 |
| dre00511 | Other glycan degradation | 4/900 | 28/8379 | 0.354270801390785 | 0.888488676503874 | 445250 445250\_1 566517 100000684\_1 | neu3.2 neu3.2 aga neu3.5 | dre:445250 dre:445250 dre:566517 dre:100000684 |
| dre00630 | Glyoxylate and dicarboxylate metabolism | 6/900 | 46/8379 | 0.372792118305104 | 0.907101119611519 | 335715 322670\_1 437019\_1 541329 541329\_1 792824\_1 | mdh1ab aco2 pcca gcsha gcsha pgp | dre:335715 dre:322670 dre:437019 dre:541329 dre:541329 dre:792824 |
| dre04510 | Focal adhesion | 40/900 | 354/8379 | 0.390327942613653 | 0.907101119611519 | 57934\_1 57934 562529\_1 30429\_2 793158 570868\_1 436718\_1 373107\_1 436718 321243\_1 563023\_1 100000252\_1 793158\_1 386856\_1 100000252\_3 100006675 30037 563023 novel.946 677750 556842\_1 571431 560564\_2 245702\_1 556226\_1 796490\_2 100005469\_1 58100\_1 378480 30429 100000252 245702 103909601 797491\_1 564565 564266 796537\_1 497384\_1 792699 101882911 | actb1 actb1 flna mylpfa cdc42l itga1 rhocb birc2 rhocb lama5 flnb pik3r3b cdc42l pdgfra pik3r3b prkcba tnc flnb - myl2b tln2a LOC571431 pdgfc igf1rb cdc42l2 pdgfbb fn1a badb pdgfaa mylpfa pik3r3b igf1rb LOC103909601 itgb7 arhgap35 itgb3a kdrl prkca rac1l LOC101882911 | dre:57934 dre:57934 dre:562529 dre:30429 dre:793158 dre:570868 dre:436718 dre:373107 dre:436718 dre:321243 dre:563023 dre:100000252 dre:793158 dre:386856 dre:100000252 dre:100006675 dre:30037 dre:563023 dre:386856 dre:677750 dre:556842 dre:571431 dre:560564 dre:245702 dre:556226 dre:796490 dre:100005469 dre:58100 dre:103909601 dre:30429 dre:100000252 dre:245702 dre:103909601 dre:797491 dre:564565 dre:564266 dre:796537 dre:497384 dre:792699 dre:321070 |
| dre00071 | Fatty acid degradation | 8/900 | 65/8379 | 0.398167619498776 | 0.907101119611519 | 100005587\_1 116517 100536795 393860\_1 368239 393860 393427 100006238\_1 | aldh9a1a.1 adh5 wu:fb14d09 gcdha aldh2.2 gcdha zgc:63568 aldh9a1a.2 | dre:100005587 dre:116517 dre:100536795 dre:393860 dre:368239 dre:393860 dre:393427 dre:100006238 |
| dre00860 | Porphyrin metabolism | 9/900 | 74/8379 | 0.399579330600468 | 0.907101119611519 | 100002875 100002875\_1 406731 101886332 100415796 570913\_1 100329531\_1 64607\_1 767756 | hmox2a hmox2a ugt1ab hephl1b ugt5b5 ugt5g1 hmox2b alas2 ugt5a2 | dre:100002875 dre:100002875 dre:406731 dre:101886332 dre:678597 dre:570913 dre:100329531 dre:64607 dre:767756 |
| dre01040 | Biosynthesis of unsaturated fatty acids | 5/900 | 39/8379 | 0.41003033252764 | 0.907101119611519 | 140615 334217 393441 554145 317738\_1 | fads2 elovl7a hacd3 elovl8b elovl6 | dre:140615 dre:334217 dre:393441 dre:554145 dre:317738 |
| dre04625 | C-type lectin receptor signaling pathway | 18/900 | 156/8379 | 0.410699963617618 | 0.907101119611519 | 436718\_1 565241\_2 336121 559020 562552 436718 100000252\_1 100000252\_3 795414\_2 368241 566185 571242\_2 556277 561021 368481 565241 100000252 561021\_1 | rhocb ppp3ccb calm2a ptgs2b zgc:171775 rhocb pik3r3b pik3r3b relb egr2a caspbl malt3 cblb si:dkey-103e21.5 stat1b ppp3ccb pik3r3b si:dkey-103e21.5 | dre:436718 dre:565241 dre:192322 dre:559020 dre:562552 dre:436718 dre:100000252 dre:100000252 dre:795414 dre:368241 dre:566185 dre:571242 dre:556277 dre:561021 dre:368481 dre:565241 dre:100000252 dre:561021 |
| dre03013 | Nucleocytoplasmic transport | 15/900 | 129/8379 | 0.413084453221689 | 0.907101119611519 | 393693\_1 100005893 393693 393973 324342\_1 100005893\_1 407710\_1 406860 324342 100004503\_1 321744\_1 100004503 407710 333980 393973\_1 | sap18 magoh sap18 rae1 xpo4 magoh xpo7 ipo9 xpo4 eef1a1b nup88 eef1a1b xpo7 xpo6 rae1 | dre:393693 dre:100005893 dre:393693 dre:393973 dre:324342 dre:100005893 dre:407710 dre:406860 dre:324342 dre:100004503 dre:321744 dre:100004503 dre:407710 dre:333980 dre:393973 |
| dre03266 | Virion - Herpesvirus | 2/900 | 13/8379 | 0.414686648054194 | 0.907101119611519 | 791704 564266 | zgc:153759 itgb3a | dre:791704 dre:564266 |
| dre04330 | Notch signaling pathway | 11/900 | 93/8379 | 0.415314444371641 | 0.907101119611519 | 678649\_1 678649 566841 393500\_1 393500 557340\_1 557340 30120\_1 497616 140422\_1 140422 | zgc:136929 zgc:136929 crebbpa zgc:66440 zgc:66440 atxn1a atxn1a dlc numbl jag2b jag2b | dre:678649 dre:678649 dre:566841 dre:393500 dre:393500 dre:557340 dre:557340 dre:30120 dre:497616 dre:140422 dre:140422 |
| dre00480 | Glutathione metabolism | 10/900 | 85/8379 | 0.430585974499139 | 0.907101119611519 | 100330864 406762\_1 553575\_1 570579\_1 100006589\_1 555317 393493 406703 431762\_1 450084\_1 | LOC100330864 pgd gsr g6pd idh1 si:ch211-276a23.5 gstt2 gsta.1 mgst1.2 gss | dre:100330864 dre:406762 dre:553575 dre:570579 dre:100006589 dre:555317 dre:393493 dre:406703 dre:431762 dre:450084 |
| dre00980 | Metabolism of xenobiotics by cytochrome P450 | 10/900 | 85/8379 | 0.430585974499139 | 0.907101119611519 | 116517 406731 393293 100415796 393493 570913\_1 406703 393427 431762\_1 767756 | adh5 ugt1ab hsd11b1la ugt5b5 gstt2 ugt5g1 gsta.1 zgc:63568 mgst1.2 ugt5a2 | dre:116517 dre:406731 dre:100006793 dre:678597 dre:393493 dre:570913 dre:406703 dre:393427 dre:431762 dre:767756 |
| dre00982 | Drug metabolism - cytochrome P450 | 10/900 | 85/8379 | 0.430585974499139 | 0.907101119611519 | 116517 406731 100415796 393493 570913\_1 406703 393427 431762\_1 794311 767756 | adh5 ugt1ab ugt5b5 gstt2 ugt5g1 gsta.1 zgc:63568 mgst1.2 fmo5 ugt5a2 | dre:116517 dre:406731 dre:678597 dre:393493 dre:570913 dre:406703 dre:393427 dre:431762 dre:794311 dre:767756 |
| dre00830 | Retinol metabolism | 12/900 | 104/8379 | 0.44222484210885 | 0.907290976672085 | 116517 562840 406731 553969 445083 393580 100415796 553239 570913\_1 393427 407663 767756 | adh5 rdh10a ugt1ab cyp3a65 dhrs3a bco1l ugt5b5 lrata ugt5g1 zgc:63568 si:ch211-107o10.3 ugt5a2 | dre:116517 dre:562840 dre:406731 dre:553969 dre:445083 dre:393580 dre:678597 dre:553239 dre:570913 dre:393427 dre:407663 dre:767756 |
| dre04621 | NOD-like receptor signaling pathway | 23/900 | 205/8379 | 0.44516564351529 | 0.907290976672085 | 100000242 436718\_1 373107\_1 393186\_1 562552 436718 557239 326974\_1 436734\_1 569298 566185 564295\_2 557239\_1 561021 323944 100334992 368481 110437957 100002946 110438502 561021\_1 403131 100149641 | zgc:92606 rhocb birc2 zgc:56235 zgc:171775 rhocb antxr2a gabarapa txn ctsbb caspbl traf2a antxr2a si:dkey-103e21.5 si:ch1073-155h21.1 unm\_sa911 stat1b LOC110437957 cxcl8a LOC110438502 si:dkey-103e21.5 tlr4ba LOC100149641 | dre:100000242 dre:436718 dre:373107 dre:393186 dre:562552 dre:436718 dre:557239 dre:326974 dre:436734 dre:569298 dre:566185 dre:564295 dre:557239 dre:561021 dre:323944 dre:100334992 dre:368481 dre:373874 dre:100002946 dre:796505 dre:561021 dre:403131 dre:100149641 |
| dre04217 | Necroptosis | 24/900 | 215/8379 | 0.452938938331619 | 0.907290976672085 | 336426\_1 559768 321622 110438871 393444 373107\_1 336426 393186\_1 337413 337413\_1 550542\_1 558816 100126128 566185 564295\_2 768248 561021 368481 100148818 100149436\_1 100149436 561021\_1 100148591 403131 | chmp1b zgc:173593 hmgb1a LOC110438871 pygmb birc2 chmp1b zgc:56235 stat6 stat6 chmp4c LOC558816 zgc:173594 caspbl traf2a fas si:dkey-103e21.5 stat1b si:ch73-55i23.1 si:ch211-189a15.5 si:ch211-189a15.5 si:dkey-103e21.5 LOC100148591 tlr4ba | dre:336426 dre:559768 dre:110438871 dre:110438871 dre:393444 dre:373107 dre:336426 dre:393186 dre:337413 dre:337413 dre:550542 dre:558816 dre:100126128 dre:566185 dre:564295 dre:768248 dre:561021 dre:368481 dre:100148818 dre:100149436 dre:100149436 dre:561021 dre:561187 dre:403131 |
| dre04146 | Peroxisome | 13/900 | 114/8379 | 0.453645488336042 | 0.907290976672085 | 100093708\_1 450048 100006589\_1 406803 562105 100093708 405800 406803\_1 406803\_2 100003383\_1 100003383 110440136 619259 | slc25a17 ech1 idh1 abcd3a si:dkey-275b16.2 slc25a17 dao.2 abcd3a abcd3a pmvk pmvk LOC110440136 dao.1 | dre:100093708 dre:450048 dre:100006589 dre:406803 dre:110440136 dre:100093708 dre:405800 dre:406803 dre:406803 dre:100003383 dre:100003383 dre:110440136 dre:619259 |
| dre00910 | Nitrogen metabolism | 3/900 | 24/8379 | 0.484448100877138 | 0.956784999232347 | 569989\_1 100006448 767680 | ca5a ca6 zgc:153760 | dre:569989 dre:100006448 dre:767680 |
| dre04068 | FoxO signaling pathway | 23/900 | 210/8379 | 0.493815075368124 | 0.9585939635807 | 324140\_1 324140 100000242 406473 431763\_1 117508\_1 431763 566841 562552 100000252\_1 117508 767751\_1 100000252\_3 326974\_1 571431 245702\_1 565576 563708 100000252 245702 100006858\_1 280649\_1 559723 | sgk1 sgk1 zgc:92606 pck2 gadd45ab klf2a gadd45ab crebbpa zgc:171775 pik3r3b klf2a ccnb3 pik3r3b gabarapa LOC571431 igf1rb plk2b skp2 pik3r3b igf1rb csnk1e plk1 tgfb2l | dre:324140 dre:324140 dre:100000242 dre:406473 dre:431763 dre:117508 dre:431763 dre:566841 dre:562552 dre:100000252 dre:117508 dre:767751 dre:100000252 dre:326974 dre:571431 dre:245702 dre:565576 dre:563708 dre:100000252 dre:245702 dre:100006858 dre:280649 dre:559723 |
| dre00280 | Valine, leucine and isoleucine degradation | 8/900 | 71/8379 | 0.499262541572016 | 0.9585939635807 | 100005587\_1 100536795 368239 437019\_1 394130\_1 797311 777632 100006238\_1 | aldh9a1a.1 wu:fb14d09 aldh2.2 pcca acad8 LOC797311 mccc1 aldh9a1a.2 | dre:100005587 dre:100536795 dre:368239 dre:437019 dre:394130 dre:797311 dre:777632 dre:100006238 |
| dre00040 | Pentose and glucuronate interconversions | 7/900 | 62/8379 | 0.504585403084278 | 0.9585939635807 | 406731 100415796 570913\_1 445326\_1 393350 445326 767756 | ugt1ab ugt5b5 ugt5g1 akr1a1a xylb akr1a1a ugt5a2 | dre:406731 dre:678597 dre:570913 dre:445326 dre:393350 dre:445326 dre:767756 |
| dre00670 | One carbon pool by folate | 3/900 | 25/8379 | 0.512572189489222 | 0.9585939635807 | 100526683\_1 557552 100004977\_2 | aldh1l1 zgc:153031 mthfd2 | dre:100526683 dre:557552 dre:100004977 |
| dre04623 | Cytosolic DNA-sensing pathway | 8/900 | 72/8379 | 0.515699284204807 | 0.9585939635807 | 140621 394162 562329 566185 394162\_1 140621\_1 561021 561021\_1 | casp3a polr3f polr3b caspbl polr3f casp3a si:dkey-103e21.5 si:dkey-103e21.5 | dre:140621 dre:394162 dre:562329 dre:566185 dre:394162 dre:140621 dre:561021 dre:561021 |
| dre04216 | Ferroptosis | 8/900 | 73/8379 | 0.53195109807225 | 0.977305505760645 | 559768 796322 393186\_1 558816 100126128 790938 450084\_1 100320152 | zgc:173593 slc3a2a zgc:56235 LOC558816 zgc:173594 zgc:158423 gss steap3 | dre:559768 dre:796322 dre:393186 dre:558816 dre:100126128 dre:790938 dre:450084 dre:100320152 |
| dre04530 | Tight junction | 32/900 | 300/8379 | 0.545588462939109 | 0.980760769489486 | 57934\_1 337226\_1 337226 57934 286739\_1 569084\_1 793158 436718\_1 286739 81585 792828 436718 60635\_1 798632 793158\_1 100536545 792828\_1 110438642 677750 556226\_1 793143 556298 797651 797651\_1 559392\_1 431777 445305\_1 793952\_1 100005566 559392 792699 101882911 | actb1 zgc:153867 zgc:153867 actb1 msna cldni cdc42l rhocb msna cldnf pard6a rhocb cldn7b myl6 cdc42l synpo pard6a LOC110438642 myl2b cdc42l2 si:dkey-98f17.3 micall2b jam3a jam3a ppp2r2cb tuba7l arpc4l si:dkey-253i9.4 f11r.2 ppp2r2cb rac1l LOC101882911 | dre:57934 dre:337226 dre:337226 dre:57934 dre:286739 dre:569084 dre:793158 dre:436718 dre:286739 dre:81585 dre:792828 dre:436718 dre:60635 dre:798632 dre:793158 dre:100536545 dre:792828 dre:110438642 dre:677750 dre:556226 dre:793143 dre:556298 dre:797651 dre:797651 dre:559392 dre:431777 dre:445305 dre:793952 dre:100005566 dre:559392 dre:792699 dre:321070 |
| dre00514 | Other types of O-glycan biosynthesis | 7/900 | 65/8379 | 0.556493833488113 | 0.980760769489486 | 556077\_1 557675 557675\_1 555344 569769 337131 568500 | plod3 c1galt1a c1galt1a si:dkey-202e17.1 pomt1 c1galt1b galnt8a.2 | dre:556077 dre:557675 dre:557675 dre:555344 dre:569769 dre:337131 dre:568500 |
| dre04540 | Gap junction | 17/900 | 160/8379 | 0.557579510683971 | 0.980760769489486 | 323509\_1 386856\_1 406811\_1 100006675 568726 novel.946 571431 560564\_2 796490\_2 378480 100000981\_2 566430\_1 431777 560807 103909601 497384\_1 559806 | gnaia pdgfra zgc:55461 prkcba adcy7 - LOC571431 pdgfc pdgfbb pdgfaa htr2cl1 prkg1b tuba7l adcy6b LOC103909601 prkca htr2ab | dre:323509 dre:386856 dre:336681 dre:100006675 dre:568726 dre:386856 dre:571431 dre:560564 dre:796490 dre:103909601 dre:100000981 dre:566430 dre:431777 dre:560807 dre:103909601 dre:497384 dre:559806 |
| dre00062 | Fatty acid elongation | 5/900 | 46/8379 | 0.559053580592027 | 0.980760769489486 | 334217 393441 554145 393657 317738\_1 | elovl7a hacd3 elovl8b zgc:66024 elovl6 | dre:334217 dre:393441 dre:554145 dre:393657 dre:317738 |
| dre04916 | Melanogenesis | 18/900 | 171/8379 | 0.573791802818922 | 0.980760769489486 | 323509\_1 336121 566841 100006675 568726 100333318 556087\_1 58072 30369\_1 791993 393760\_1 30127\_1 560807 100538072 556087 553181\_1 58072\_1 497384\_1 | gnaia calm2a crebbpa prkcba adcy7 creb3l4 wnt2bb fzd8b fzd7a wnt4b gnao1a wnt2 adcy6b creb3l3b wnt2bb kitlgb fzd8b prkca | dre:323509 dre:192322 dre:566841 dre:100006675 dre:568726 dre:100333318 dre:556087 dre:58072 dre:30369 dre:791993 dre:393760 dre:30127 dre:560807 dre:100538072 dre:556087 dre:553181 dre:58072 dre:497384 |
| dre00250 | Alanine, aspartate and glutamate metabolism | 6/900 | 57/8379 | 0.584457819408786 | 0.980760769489486 | 447815\_1 334431\_1 447815 564754\_1 565235\_1 406688 | rimkla adsl rimkla nat8l aldh5a1 got2a | dre:447815 dre:334431 dre:447815 dre:564754 dre:565235 dre:406688 |
| dre00730 | Thiamine metabolism | 2/900 | 18/8379 | 0.590772632396283 | 0.980760769489486 | 100330410 574009 | alp3 alpi.2 | dre:100330410 dre:574009 |
| dre00770 | Pantothenate and CoA biosynthesis | 3/900 | 28/8379 | 0.591683268387111 | 0.980760769489486 | 100536795 368239 406789 | wu:fb14d09 aldh2.2 pank1a | dre:100536795 dre:368239 dre:406789 |
| dre00790 | Folate biosynthesis | 4/900 | 38/8379 | 0.594576816698717 | 0.980760769489486 | 557552 791448\_1 100330410 574009 | zgc:153031 qdprb1 alp3 alpi.2 | dre:557552 dre:791448 dre:100330410 dre:574009 |
| dre04520 | Adherens junction | 20/900 | 194/8379 | 0.612910416986685 | 0.980760769489486 | 57934\_1 57934 30429\_2 793158 436718\_1 566841 436718 793158\_1 677750 569566 335573\_1 571431 245702\_1 556226\_1 393527 30429 245702 564805\_1 792699 101882911 | actb1 actb1 mylpfa cdc42l rhocb crebbpa rhocb cdc42l myl2b LOC569566 ptpn6 LOC571431 igf1rb cdc42l2 pdcd10a mylpfa igf1rb arhgap29a rac1l LOC101882911 | dre:57934 dre:57934 dre:30429 dre:793158 dre:436718 dre:566841 dre:436718 dre:793158 dre:677750 dre:569566 dre:335573 dre:571431 dre:245702 dre:556226 dre:393527 dre:30429 dre:245702 dre:378998 dre:792699 dre:321070 |
| dre04142 | Lysosome | 22/900 | 214/8379 | 0.620633584209805 | 0.980760769489486 | 565099 571066 100003043\_1 406511 553423\_1 569298 337771 450083 368911 436725\_1 553423 566517 445244 436725 559575 565246 327237\_1 570148\_1 406511\_1 393657 445244\_1 567720 | zgc:193725 si:ch1073-513e17.1 lamp3 sort1a sumf1 ctsbb zgc:162858 gla laptm4b acp5b sumf1 aga ap1m3 acp5b si:ch211-15d5.11 si:dkey-192p21.6 ap1s2 abcb9 sort1a zgc:66024 ap1m3 idua | dre:565099 dre:571066 dre:100003043 dre:406511 dre:553423 dre:569298 dre:337771 dre:450083 dre:368911 dre:436725 dre:553423 dre:566517 dre:445244 dre:436725 dre:559575 dre:565246 dre:327237 dre:570148 dre:406511 dre:393657 dre:445244 dre:567720 |
| dre00604 | Glycosphingolipid biosynthesis - ganglio series | 2/900 | 19/8379 | 0.620897816024102 | 0.980760769489486 | 394230 394230\_1 | st3gal5 st3gal5 | dre:394230 dre:394230 |
| dre04261 | Adrenergic signaling in cardiomyocytes | 31/900 | 301/8379 | 0.62798258139163 | 0.980760769489486 | 323509\_1 336121 64614 562552 556410\_1 793666\_1 100535196 568726 555423\_1 677750 100333318 100037358\_1 793666 559015 794522 678560\_1 562282\_1 555423 559392\_1 562282 492486\_1 114457\_1 353247 550357\_2 560807 492486 100538072 114457 559392 558525\_1 497384\_1 | gnaia calm2a atp1a1a.3 zgc:171775 atf4b popdc2 cacna2d3a adcy7 scn1ba myl2b creb3l4 zgc:163073 popdc2 cacnb3b si:ch211-139a5.9 cacnb4b ppp2r5b scn1ba ppp2r2cb ppp2r5b adra1bb atp1b2b tnnc1a cremb adcy6b adra1bb creb3l3b atp1b2b ppp2r2cb atp2b3b prkca | dre:323509 dre:192322 dre:64614 dre:562552 dre:556410 dre:793666 dre:100535196 dre:568726 dre:555423 dre:677750 dre:100333318 dre:100037358 dre:793666 dre:559015 dre:794522 dre:678560 dre:562282 dre:555423 dre:559392 dre:562282 dre:492486 dre:114457 dre:353247 dre:550357 dre:560807 dre:492486 dre:100538072 dre:114457 dre:559392 dre:558525 dre:497384 |
| dre04912 | GnRH signaling pathway | 16/900 | 158/8379 | 0.637922392497142 | 0.980760769489486 | 793158 336121 30498\_1 562552 556410\_1 793158\_1 566945 100006675 30498 568726 571431 556226\_1 565743\_1 100148818 560807 497384\_1 | cdc42l calm2a egr1 zgc:171775 atf4b cdc42l mmp14b prkcba egr1 adcy7 LOC571431 cdc42l2 pld2 si:ch73-55i23.1 adcy6b prkca | dre:793158 dre:192322 dre:30498 dre:562552 dre:556410 dre:793158 dre:566945 dre:100006675 dre:30498 dre:568726 dre:571431 dre:556226 dre:565743 dre:100148818 dre:560807 dre:497384 |
| dre04270 | Vascular smooth muscle contraction | 23/900 | 226/8379 | 0.641753623806402 | 0.980760769489486 | 337226\_1 337226 436718\_1 336121 406370\_1 436718 798632 406370 100006675 436966\_2 568726 403114\_1 567131 436966 403114 492486\_1 566430\_1 100148818 393783\_1 560807 492486 497384\_1 101882911 | zgc:153867 zgc:153867 rhocb calm2a pla2g6 rhocb myl6 pla2g6 prkcba zgc:91818 adcy7 adm2a calcrlb zgc:91818 adm2a adra1bb prkg1b si:ch73-55i23.1 ppp1r14ab adcy6b adra1bb prkca LOC101882911 | dre:337226 dre:337226 dre:436718 dre:192322 dre:406370 dre:436718 dre:798632 dre:406370 dre:100006675 dre:436966 dre:568726 dre:403114 dre:567131 dre:436966 dre:403114 dre:492486 dre:566430 dre:100148818 dre:393783 dre:560807 dre:492486 dre:497384 dre:321070 |
| dre01250 | Biosynthesis of nucleotide sugars | 5/900 | 51/8379 | 0.652959937757126 | 0.980760769489486 | 406339\_1 100007686 100885772 321224 751668 | hk2 LOC100007686 cmasb hkdc1 gck | dre:406339 dre:100007686 dre:100885772 dre:321224 dre:751668 |
| dre04136 | Autophagy - other | 4/900 | 41/8379 | 0.655740257795878 | 0.980760769489486 | 100000242 792689\_1 326974\_1 406843\_1 | zgc:92606 mlst8 gabarapa igbp1 | dre:100000242 dre:792689 dre:326974 dre:406843 |
| dre04620 | Toll-like receptor signaling pathway | 12/900 | 121/8379 | 0.659609741372567 | 0.980760769489486 | 562552 100000252\_1 100000252\_3 394198 570238 561021 368481 100002946 100000252 561021\_1 403131 792699 | zgc:171775 pik3r3b pik3r3b fosab fosaa si:dkey-103e21.5 stat1b cxcl8a pik3r3b si:dkey-103e21.5 tlr4ba rac1l | dre:562552 dre:100000252 dre:100000252 dre:394198 dre:570238 dre:561021 dre:368481 dre:100002946 dre:100000252 dre:561021 dre:403131 dre:792699 |
| dre04145 | Phagosome | 21/900 | 209/8379 | 0.661929757708319 | 0.980760769489486 | 57934\_1 57934 359840\_1 406811\_1 359840 100004793 30762 571584 368878 100537272 403014 100005358 30763 431777 359839 571282 403131 564266 556826\_1 792699 100007982 | actb1 actb1 atp6v1b2 zgc:55461 atp6v1b2 mrc2 mhc2dab marco si:busm1-266f07.2 LOC100537272 ncf4 si:ch211-274f20.2 mhc2dcb tuba7l atp6v1ba LOC571282 tlr4ba itgb3a si:dkey-57h18.2 rac1l hbl1 | dre:57934 dre:57934 dre:359840 dre:336681 dre:359840 dre:100004793 dre:30762 dre:571584 dre:368878 dre:100537272 dre:403014 dre:100005358 dre:30763 dre:431777 dre:359839 dre:571282 dre:403131 dre:564266 dre:556826 dre:792699 dre:100007982 |
| dre00120 | Primary bile acid biosynthesis | 3/900 | 31/8379 | 0.661993138757388 | 0.980760769489486 | 723999 641477 795106 | zgc:136333 zgc:123299 cyp27a1.2 | dre:795106 dre:553543 dre:795106 |
| dre03020 | RNA polymerase | 4/900 | 42/8379 | 0.674690749817135 | 0.980760769489486 | 394162 325603 562329 394162\_1 | polr3f polr1b polr3b polr3f | dre:394162 dre:325603 dre:562329 dre:394162 |
| dre04914 | Progesterone-mediated oocyte maturation | 13/900 | 133/8379 | 0.683147443931525 | 0.980760769489486 | 323509\_1 562552 100000252\_1 767751\_1 100000252\_3 568726 245702\_1 100000252 393223\_1 560807 245702 100009657\_1 280649\_1 | gnaia zgc:171775 pik3r3b ccnb3 pik3r3b adcy7 igf1rb pik3r3b fzr1a adcy6b igf1rb rps6ka3b plk1 | dre:323509 dre:562552 dre:100000252 dre:767751 dre:100000252 dre:568726 dre:245702 dre:100000252 dre:393223 dre:560807 dre:245702 dre:100009657 dre:280649 |
| dre04140 | Autophagy - animal | 24/900 | 241/8379 | 0.68575137620208 | 0.980760769489486 | 321622 100000242 110438871 792229\_1 792229 449923 100000252\_1 573492 792689\_1 100000252\_3 326974\_1 567021 449923\_1 569298 245702\_1 100330617\_1 58100\_1 559564 563140 406843\_1 563140\_1 100000252 245702 573492\_1 | hmgb1a zgc:92606 LOC110438871 sh3glb1a sh3glb1a si:ch73-234b20.5 pik3r3b rragca mlst8 pik3r3b gabarapa ern2 si:ch73-234b20.5 ctsbb igf1rb tsc1b badb eif2ak3 si:ch211-11n16.2 igbp1 si:ch211-11n16.2 pik3r3b igf1rb rragca | dre:110438871 dre:100000242 dre:110438871 dre:792229 dre:792229 dre:449923 dre:100000252 dre:573492 dre:792689 dre:100000252 dre:326974 dre:567021 dre:449923 dre:569298 dre:245702 dre:100330617 dre:58100 dre:559564 dre:563140 dre:406843 dre:563140 dre:100000252 dre:245702 dre:573492 |
| dre04514 | Cell adhesion molecules | 20/900 | 204/8379 | 0.702582644584545 | 0.980760769489486 | 569084\_1 30194 81585 60635\_1 557935 110438642 30762 793143 30634\_1 368878 797651 797651\_1 30763 571282 101886406 541474 797491\_1 100151365\_1 100005566 562934\_1 | cldni alcama cldnf cldn7b zmp:0000001082 LOC110438642 mhc2dab si:dkey-98f17.3 nadl1.2 si:busm1-266f07.2 jam3a jam3a mhc2dcb LOC571282 cd28 cntn1b itgb7 si:dkey-1h24.6 f11r.2 esamb | dre:569084 dre:30194 dre:81585 dre:60635 dre:557935 dre:110438642 dre:30762 dre:793143 dre:30634 dre:368878 dre:797651 dre:797651 dre:30763 dre:571282 dre:101886406 dre:541474 dre:797491 dre:100151365 dre:100005566 dre:562934 |
| dre00564 | Glycerophospholipid metabolism | 13/900 | 135/8379 | 0.70383533802471 | 0.980760769489486 | 724010 100002015\_1 724010\_1 406370\_1 406370 799099\_1 334017\_1 565743\_1 560225 560649 767688 100148818 572841 | dgkaa cds1 dgkaa pla2g6 pla2g6 lpin1 pla1a pld2 cept1a si:ch211-214p16.3 zgc:154040 si:ch73-55i23.1 mboat1 | dre:724010 dre:100002015 dre:724010 dre:406370 dre:406370 dre:799099 dre:334017 dre:565743 dre:560225 dre:560649 dre:767688 dre:100148818 dre:572841 |
| dre04622 | RIG-I-like receptor signaling pathway | 7/900 | 76/8379 | 0.721731763702853 | 0.980760769489486 | 562552 564295\_2 561021 100002946 393144\_2 100148871 561021\_1 | zgc:171775 traf2a si:dkey-103e21.5 cxcl8a trim25 dhx58 si:dkey-103e21.5 | dre:562552 dre:564295 dre:561021 dre:100002946 dre:393144 dre:100148871 dre:561021 |
| dre03022 | Basal transcription factors | 4/900 | 45/8379 | 0.727120927798225 | 0.980760769489486 | 798997\_2 798997\_1 567899 798997 | gtf2a2 gtf2a2 gtf2e1 gtf2a2 | dre:798997 dre:798997 dre:567899 dre:798997 |
| dre04310 | Wnt signaling pathway | 27/900 | 278/8379 | 0.741584653927604 | 0.980760769489486 | 436718\_1 565241\_2 678649\_1 678649 566841 436718 323883 100000500\_1 100006675 556087\_1 58072 30369\_1 564241 103909653 791993 565241 402843\_1 30127\_1 30197 556087 58072\_1 100006858\_1 798564 326875\_1 497384\_1 792699 101882911 | rhocb ppp3ccb zgc:136929 zgc:136929 crebbpa rhocb cul1a sost prkcba wnt2bb fzd8b fzd7a fosl1a LOC103909653 wnt4b ppp3ccb sfrp1a wnt2 dkk1b wnt2bb fzd8b csnk1e sfrp1b cby1 prkca rac1l LOC101882911 | dre:436718 dre:565241 dre:678649 dre:678649 dre:566841 dre:436718 dre:323883 dre:100000500 dre:100006675 dre:556087 dre:58072 dre:30369 dre:564241 dre:103909653 dre:791993 dre:565241 dre:402843 dre:30127 dre:30197 dre:556087 dre:58072 dre:100006858 dre:798564 dre:326875 dre:497384 dre:792699 dre:321070 |
| dre03420 | Nucleotide excision repair | 6/900 | 68/8379 | 0.752734707509833 | 0.980760769489486 | 393774\_2 393774 556995\_1 556995\_2 553405\_1 100006257 | pole3 pole3 lig1 lig1 pole cetn2 | dre:393774 dre:393774 dre:556995 dre:556995 dre:553405 dre:100006257 |
| dre04910 | Insulin signaling pathway | 20/900 | 212/8379 | 0.764849207572595 | 0.980760769489486 | 100001260\_1 393444 406473 336121 100001260 79380 100000252\_1 79380\_1 100000252\_3 406339\_1 556277 100330617\_1 58100\_1 559403\_1 100000252 559001\_1 321224 751668 406459 569706\_1 | ppp1r3ab pygmb pck2 calm2a ppp1r3ab eif4ea pik3r3b eif4ea pik3r3b hk2 cblb tsc1b badb acaca pik3r3b fasn hkdc1 gck zgc:77112 phkb | dre:100001260 dre:393444 dre:406473 dre:192322 dre:100001260 dre:79380 dre:100000252 dre:79380 dre:100000252 dre:406339 dre:556277 dre:100330617 dre:58100 dre:559403 dre:100000252 dre:559001 dre:321224 dre:751668 dre:406459 dre:569706 |
| dre04060 | Cytokine-cytokine receptor interaction | 23/900 | 242/8379 | 0.765464426805778 | 0.980760769489486 | 799354 368326\_1 58151\_1 561050\_1 799279 368438\_1 100037357 799354\_1 368326 562247 768248 100006078\_1 100135755 562247\_1 58133 100002946 791704 100006078 100135062\_2 100001424 100333914 30125 559723 | cxcl12b il13ra2 cxcl14 ackr3b LOC799279 cntfr tnfrsf1b cxcl12b il13ra2 clcf1 fas ccr9b il6r clcf1 ngfb cxcl8a zgc:153759 ccr9b csf2rb ghra ccl25a gdf3 tgfb2l | dre:799354 dre:368326 dre:58151 dre:561050 dre:799279 dre:368438 dre:100037357 dre:799354 dre:368326 dre:562247 dre:768248 dre:100006078 dre:100135755 dre:562247 dre:58133 dre:100002946 dre:791704 dre:100006078 dre:100135062 dre:100001424 dre:100333914 dre:30125 dre:559723 |
| dre00563 | Glycosylphosphatidylinositol (GPI)-anchor biosynthesis | 2/900 | 25/8379 | 0.766422931210333 | 0.980760769489486 | 561471 561471\_1 | pigl pigl | dre:561471 dre:561471 |
| dre04020 | Calcium signaling pathway | 44/900 | 450/8379 | 0.77303987572048 | 0.980760769489486 | 565241\_2 336121 393186\_1 368717 368717\_1 544664 386856\_1 544664\_1 100006675 568726 566671 100005049 novel.946 569566 571431 560564\_2 387296\_1 796490\_2 494456 58147 58147\_1 378480 492486\_1 100000981\_2 101886891 565241 58133 353247 259258 492486 794365 100005590 103909601 445226 558525\_1 796537\_1 100005590\_1 497384\_1 555941 559806 100137117 569706\_1 570493\_1 100137117\_1 | ppp3ccb calm2a zgc:56235 itpka itpka erbb3b pdgfra erbb3b prkcba adcy7 orai1a fgf1b - LOC569566 LOC571431 pdgfc p2rx1 pdgfbb fgf23 p2rx3a p2rx3a pdgfaa adra1bb htr2cl1 LOC101886891 ppp3ccb ngfb tnnc1a p2rx4a adra1bb si:dkey-163m14.7 hrh2b LOC103909601 casq1a atp2b3b kdrl hrh2b prkca cacna1ba htr2ab si:dkey-1h24.2 phkb grin2ab si:dkey-1h24.2 | dre:565241 dre:192322 dre:393186 dre:368717 dre:368717 dre:544664 dre:386856 dre:100149258 dre:100006675 dre:568726 dre:566671 dre:100005049 dre:386856 dre:569566 dre:571431 dre:560564 dre:387296 dre:796490 dre:494456 dre:58147 dre:58147 dre:103909601 dre:492486 dre:100000981 dre:101886891 dre:565241 dre:58133 dre:353247 dre:259258 dre:492486 dre:794365 dre:100005590 dre:103909601 dre:445226 dre:558525 dre:796537 dre:100005590 dre:497384 dre:555941 dre:559806 dre:100137117 dre:569706 dre:570493 dre:100137117 |
| dre04260 | Cardiac muscle contraction | 16/900 | 173/8379 | 0.773902250019496 | 0.980760769489486 | 393793\_1 64614 447942\_1 100535196 447942 677750 556638\_2 100037358\_1 559015 794522 678560\_1 114457\_1 353247 556638\_1 114457 436768 | uqcrc1 atp1a1a.3 cox6a2 cacna2d3a cox6a2 myl2b slc9a6b zgc:163073 cacnb3b si:ch211-139a5.9 cacnb4b atp1b2b tnnc1a slc9a6b atp1b2b uqcrq | dre:393793 dre:64614 dre:447942 dre:100535196 dre:447942 dre:677750 dre:556638 dre:100037358 dre:559015 dre:794522 dre:678560 dre:114457 dre:353247 dre:556638 dre:114457 dre:436768 |
| dre01212 | Fatty acid metabolism | 8/900 | 91/8379 | 0.775342813778073 | 0.980760769489486 | 140615 334217 393441 559403\_1 554145 559001\_1 393657 317738\_1 | fads2 elovl7a hacd3 acaca elovl8b fasn zgc:66024 elovl6 | dre:140615 dre:334217 dre:393441 dre:559403 dre:554145 dre:559001 dre:393657 dre:317738 |
| dre00650 | Butanoate metabolism | 2/900 | 26/8379 | 0.785303219398353 | 0.980760769489486 | 797311 565235\_1 | LOC797311 aldh5a1 | dre:797311 dre:565235 |
| dre03008 | Ribosome biogenesis in eukaryotes | 7/900 | 83/8379 | 0.802123708829879 | 0.980760769489486 | 393107 407084 393107\_2 407084\_1 393107\_1 492761\_1 553787\_1 | utp6 riok2 utp6 riok2 utp6 tbl3 pop4 | dre:393107 dre:407084 dre:393107 dre:407084 dre:393107 dre:492761 dre:553787 |
| dre00531 | Glycosaminoglycan degradation | 2/900 | 27/8379 | 0.802827779482078 | 0.980760769489486 | 565246 567720 | si:dkey-192p21.6 idua | dre:565246 dre:567720 |
| dre04110 | Cell cycle | 21/900 | 229/8379 | 0.810929557969527 | 0.980760769489486 | 560751\_1 431763\_1 431763 566841 323299\_2 323883 327226 767751\_1 554509\_1 novel.902 797406\_2 563708 100141368\_1 324475\_1 562282\_1 794108\_1 562282 393223\_1 327060 280649\_1 559723 | stag2b gadd45ab gadd45ab crebbpa atrx cul1a casc5 ccnb3 orc1 - espl1 skp2 waplb smc3 ppp2r5b nipblb ppp2r5b fzr1a wee1 plk1 tgfb2l | dre:560751 dre:431763 dre:431763 dre:566841 dre:323299 dre:323883 dre:327226 dre:767751 dre:554509 dre:323299 dre:797406 dre:563708 dre:100141368 dre:324475 dre:562282 dre:794108 dre:562282 dre:393223 dre:327060 dre:280649 dre:559723 |
| dre00970 | Aminoacyl-tRNA biosynthesis | 4/900 | 51/8379 | 0.812546783425365 | 0.980760769489486 | 324940\_1 394188 101884223 792787 | aars qars LOC101884223 aars2 | dre:792787 dre:394188 dre:101884223 dre:792787 |
| dre00061 | Fatty acid biosynthesis | 2/900 | 28/8379 | 0.819069664950921 | 0.980760769489486 | 559403\_1 559001\_1 | acaca fasn | dre:559403 dre:559001 |
| dre00520 | Amino sugar and nucleotide sugar metabolism | 6/900 | 74/8379 | 0.820658946274538 | 0.980760769489486 | 406339\_1 100007686 100885772 563138 321224 751668 | hk2 LOC100007686 cmasb si:ch211-219a15.4 hkdc1 gck | dre:406339 dre:100007686 dre:100885772 dre:563138 dre:321224 dre:751668 |
| dre00640 | Propanoate metabolism | 3/900 | 41/8379 | 0.831893214845198 | 0.980760769489486 | 437019\_1 317746 559403\_1 | pcca suclg2 acaca | dre:437019 dre:317746 dre:559403 |
| dre03010 | Ribosome | 14/900 | 161/8379 | 0.835116754710154 | 0.980760769489486 | 336712\_2 415196\_1 327089 550564\_1 768187\_1 553284 336712 554098\_2 553284\_1 450009\_1 560828\_1 553799 337007 337007\_1 | rpl10 rpl5b rpl12 faub mrpl18 mrpl1 rpl10 mrpl28 mrpl1 mrps16 rpl13a mrpl30 rps12 rps12 | dre:336712 dre:415196 dre:327089 dre:550564 dre:768187 dre:553284 dre:336712 dre:554098 dre:553284 dre:450009 dre:560828 dre:553799 dre:337007 dre:337007 |
| dre04114 | Oocyte meiosis | 16/900 | 182/8379 | 0.836445555818075 | 0.980760769489486 | 565241\_2 336121 562552 323883 568726 797406\_2 245702\_1 324475\_1 562282\_1 562282 565241 560807 245702 100009657\_1 100005148\_1 280649\_1 | ppp3ccb calm2a zgc:171775 cul1a adcy7 espl1 igf1rb smc3 ppp2r5b ppp2r5b ppp3ccb adcy6b igf1rb rps6ka3b ar plk1 | dre:565241 dre:192322 dre:562552 dre:323883 dre:568726 dre:797406 dre:245702 dre:324475 dre:562282 dre:562282 dre:565241 dre:560807 dre:245702 dre:100009657 dre:100005148 dre:280649 |
| dre03450 | Non-homologous end-joining | 1/900 | 16/8379 | 0.837941416574285 | 0.980760769489486 | 566285\_1 | dclre1c | dre:566285 |
| dre04218 | Cellular senescence | 21/900 | 234/8379 | 0.839631078704557 | 0.980760769489486 | 333941\_1 565241\_2 431763\_1 336121 393186\_1 431763 562552 100000252\_1 767751\_1 100000252\_3 393176\_1 393176 557551\_1 557551 100330617\_1 100037341 565241 100002946 100000252 572042 559723 | zfp36l2 ppp3ccb gadd45ab calm2a zgc:56235 gadd45ab zgc:171775 pik3r3b ccnb3 pik3r3b rad9a rad9a hipk2 hipk2 tsc1b zgc:162895 ppp3ccb cxcl8a pik3r3b hipk1a tgfb2l | dre:333941 dre:565241 dre:431763 dre:192322 dre:393186 dre:431763 dre:562552 dre:100000252 dre:767751 dre:100000252 dre:393176 dre:393176 dre:557551 dre:557551 dre:100330617 dre:100037341 dre:565241 dre:100002946 dre:100000252 dre:572042 dre:559723 |
| dre03050 | Proteasome | 5/900 | 65/8379 | 0.841693761783163 | 0.980760769489486 | 30387 796450\_1 326884\_1 796450 436816 | psmb5 adrm1 psmc4 adrm1 zgc:92791 | dre:30387 dre:796450 dre:326884 dre:796450 dre:436816 |
| dre03040 | Spliceosome | 14/900 | 164/8379 | 0.853899821194627 | 0.980760769489486 | 100004402\_1 100037380\_3 573376 100004402 100005893 100005893\_1 566017 100037380\_2 560210 394058\_1 566017\_1 406288\_1 334283\_2 286774\_1 | plrg1 zgc:163098 hspa8 plrg1 magoh magoh lsm8 zgc:163098 hsp70l fus lsm8 srsf1a ddx23 phf5a | dre:100004402 dre:100037380 dre:573376 dre:100004402 dre:100005893 dre:100005893 dre:566017 dre:100037380 dre:30671 dre:394058 dre:566017 dre:406288 dre:334283 dre:286774 |
| dre00600 | Sphingolipid metabolism | 7/900 | 89/8379 | 0.855793754684077 | 0.980760769489486 | 445250 445250\_1 450083 100330155 100000684\_1 553981\_1 493602 | neu3.2 neu3.2 gla cers4b neu3.5 sptlc1 asah2 | dre:445250 dre:445250 dre:450083 dre:100330155 dre:100000684 dre:553981 dre:493602 |
| dre00532 | Glycosaminoglycan biosynthesis - chondroitin sulfate / dermatan sulfate | 2/900 | 31/8379 | 0.860824434650147 | 0.980760769489486 | 100331876 100538167 | LOC100331876 si:dkey-26i13.6 | dre:100331876 dre:100538167 |
| dre00140 | Steroid hormone biosynthesis | 7/900 | 91/8379 | 0.870820685524137 | 0.980760769489486 | 406731 393293 553969 100415796 570913\_1 767756 791578\_1 | ugt1ab hsd11b1la cyp3a65 ugt5b5 ugt5g1 ugt5a2 dhrs11a | dre:406731 dre:100006793 dre:553969 dre:678597 dre:570913 dre:767756 dre:791578 |
| dre04141 | Protein processing in endoplasmic reticulum | 21/900 | 241/8379 | 0.874219771656657 | 0.980760769489486 | 327197 386590\_1 386590 327197\_1 266757 266757\_1 573376 337190 567029\_1 566876 323883 556410\_1 565338\_1 567021 108181182 560210 564295\_2 100005358 559564 574002 393877 | vcp hsp90b1 hsp90b1 vcp tram1 tram1 hspa8 ssr3 rrbp1b wfs1a cul1a atf4b eif2ak1 ern2 LOC108181182 hsp70l traf2a si:ch211-274f20.2 eif2ak3 zgc:114174 derl3 | dre:327197 dre:386590 dre:386590 dre:327197 dre:266757 dre:266757 dre:573376 dre:337190 dre:567029 dre:566876 dre:323883 dre:556410 dre:565338 dre:567021 dre:567029 dre:30671 dre:564295 dre:100005358 dre:559564 dre:574002 dre:393877 |
| dre04744 | Phototransduction | 4/900 | 57/8379 | 0.874752763174483 | 0.980760769489486 | 336121 100330921 792319 140425 | calm2a si:ch211-103a14.5 saga gc2 | dre:192322 dre:100330921 dre:792319 dre:140425 |
| dre04144 | Endocytosis | 38/900 | 417/8379 | 0.883316999781842 | 0.980760769489486 | 474327\_1 336426\_1 793158 792229\_1 573376 436718\_1 792229 336426 406837\_1 406684 792828 100003902\_1 436718 550542\_1 406837 793158\_1 336368 386856\_1 393881\_1 406684\_1 387529 792828\_1 393881 560210 474327 novel.946 571431 245702\_1 568170\_2 100151129 556226\_1 556277 565743\_1 492487 568170\_1 445305\_1 561771 245702 | capza1b chmp1b cdc42l sh3glb1a hspa8 rhocb sh3glb1a chmp1b rnf41 vps26b pard6a si:dkey-16l2.16 rhocb chmp4c rnf41 cdc42l vps37a pdgfra vps26a vps26b ldlra pard6a vps26a hsp70l capza1b - LOC571431 igf1rb eps15l1a rab11fip4b cdc42l2 cblb pld2 rab11a eps15l1a arpc4l acap1 igf1rb | dre:474327 dre:336426 dre:793158 dre:792229 dre:573376 dre:436718 dre:792229 dre:336426 dre:406837 dre:406684 dre:792828 dre:100003902 dre:436718 dre:550542 dre:406837 dre:793158 dre:336368 dre:386856 dre:393881 dre:406684 dre:387529 dre:792828 dre:393881 dre:30671 dre:474327 dre:386856 dre:571431 dre:245702 dre:568170 dre:100151129 dre:556226 dre:556277 dre:565743 dre:492487 dre:568170 dre:445305 dre:561771 dre:245702 |
| dre04012 | ErbB signaling pathway | 10/900 | 127/8379 | 0.888137147770288 | 0.980760769489486 | 100000252\_1 544664 544664\_1 100000252\_3 100006675 571431 556277 58100\_1 100000252 497384\_1 | pik3r3b erbb3b erbb3b pik3r3b prkcba LOC571431 cblb badb pik3r3b prkca | dre:100000252 dre:544664 dre:100149258 dre:100000252 dre:100006675 dre:571431 dre:556277 dre:58100 dre:100000252 dre:497384 |
| dre04512 | ECM-receptor interaction | 10/900 | 128/8379 | 0.893564234794448 | 0.980760769489486 | 563428 570868\_1 563428\_1 321243\_1 30037 798998 322012\_1 100005469\_1 797491\_1 564266 | fras1 itga1 fras1 lama5 tnc gp1bb hmmr fn1a itgb7 itgb3a | dre:563428 dre:570868 dre:563428 dre:321243 dre:30037 dre:798998 dre:322012 dre:100005469 dre:797491 dre:564266 |
| dre03250 | Viral life cycle - HIV-1 | 6/900 | 83/8379 | 0.893857916496747 | 0.980760769489486 | 566841 64275\_1 559677\_1 321602\_1 562808\_1 559032\_1 | crebbpa supt5h nelfa cdk9 map1sa map1b | dre:566841 dre:64275 dre:559677 dre:321602 dre:562808 dre:559032 |
| dre00534 | Glycosaminoglycan biosynthesis - heparan sulfate / heparin | 2/900 | 38/8379 | 0.926176563936696 | 0.991411598909383 | 792524 791107\_1 | hs6st3a hs6st1b | dre:792524 dre:791107 |
| dre00430 | Taurine and hypotaurine metabolism | 1/900 | 23/8379 | 0.926987054975489 | 0.991411598909383 | 794311 | fmo5 | dre:794311 |
| dre00760 | Nicotinate and nicotinamide metabolism | 3/900 | 52/8379 | 0.928752970268397 | 0.991411598909383 | 402953\_1 101886891 110438097 | pnp6 LOC101886891 LOC110438097 | dre:402953 dre:101886891 dre:572070 |
| dre00515 | Mannose type O-glycan biosynthesis | 2/900 | 39/8379 | 0.932711334620493 | 0.991411598909383 | 569769 100093713 | pomt1 zgc:165519 | dre:569769 dre:100093713 |
| dre04371 | Apelin signaling pathway | 18/900 | 226/8379 | 0.934938786313279 | 0.991411598909383 | 323509\_1 117508\_1 336121 30498\_1 117508 30498 321449 100331581 568726 677750 100331581\_1 335655 100037358\_1 321449\_1 560807 557486\_1 335655\_1 557486 | gnaia klf2a calm2a egr1 klf2a egr1 ctgfa si:dkey-44g17.6 adcy7 myl2b si:dkey-44g17.6 gngt2a zgc:163073 ctgfa adcy6b hdac5 gngt2a hdac5 | dre:323509 dre:117508 dre:192322 dre:30498 dre:117508 dre:30498 dre:321449 dre:100331581 dre:568726 dre:677750 dre:100331581 dre:335655 dre:100037358 dre:321449 dre:560807 dre:557486 dre:335655 dre:557486 |
| dre00230 | Purine metabolism | 14/900 | 192/8379 | 0.959577875448959 | 0.999573419006275 | 100330864 334431\_1 560827\_1 777719 568726 550560\_1 553639\_1 402953\_1 333997 560807 100005067\_1 447905 140425 333997\_1 | LOC100330864 adsl prps1b papss2a adcy7 entpd6 gmpr pnp6 ampd3b adcy6b dguok entpd2a.1 gc2 ampd3b | dre:100330864 dre:334431 dre:560827 dre:777719 dre:568726 dre:550560 dre:553639 dre:402953 dre:333997 dre:560807 dre:100005067 dre:447905 dre:140425 dre:333997 |
| dre00190 | Oxidative phosphorylation | 12/900 | 171/8379 | 0.963265590731334 | 0.999573419006275 | 393793\_1 447914\_1 573619 359840\_1 447942\_1 359840 767750\_2 447942 767750 796997\_1 359839 436768 | uqcrc1 cox17 ndufab1a atp6v1b2 cox6a2 atp6v1b2 atp5ib cox6a2 atp5ib ndufs7 atp6v1ba uqcrq | dre:393793 dre:447914 dre:573619 dre:359840 dre:447942 dre:359840 dre:767750 dre:447942 dre:767750 dre:796997 dre:359839 dre:436768 |
| dre04814 | Motor proteins | 23/900 | 305/8379 | 0.977664382473915 | 0.999573419006275 | 57934\_1 337226\_1 474327\_1 337226 57934 30429\_2 334274 100149148 798632 406811\_1 101882034 474327 677750 100037358\_1 556023\_1 799461\_2 353247 431777 556023 30429 571487 562052\_1 100334351 | actb1 zgc:153867 capza1b zgc:153867 actb1 mylpfa myhc4 myha myl6 zgc:55461 LOC101882034 capza1b myl2b zgc:163073 dnah2 dnah12 tnnc1a tuba7l dnah2 mylpfa si:dkeyp-92c9.3 kif14 dnah3 | dre:57934 dre:337226 dre:474327 dre:337226 dre:57934 dre:30429 dre:334274 dre:100149148 dre:798632 dre:336681 dre:406297 dre:474327 dre:677750 dre:100037358 dre:556023 dre:799461 dre:353247 dre:431777 dre:556023 dre:30429 dre:571487 dre:562052 dre:100334351 |
| dre00220 | Arginine biosynthesis | 1/900 | 35/8379 | 0.981418746150599 | 0.999573419006275 | 406688 | got2a | dre:406688 |
| dre00601 | Glycosphingolipid biosynthesis - lacto and neolacto series | 1/900 | 42/8379 | 0.991644599390283 | 0.999573419006275 | 100093713 | zgc:165519 | dre:100093713 |
| dre04340 | Hedgehog signaling pathway | 3/900 | 79/8379 | 0.993221065075456 | 0.999573419006275 | 323883 30269\_1 100006858\_1 | cul1a shha csnk1e | dre:323883 dre:30269 dre:100006858 |
| dre04350 | TGF-beta signaling pathway | 8/900 | 161/8379 | 0.997105939931559 | 0.999573419006275 | 436718\_1 566841 436718 323883 100004116\_1 405778 556634 559723 | rhocb crebbpa rhocb cul1a fsta grem1a adamtsl7 tgfb2l | dre:436718 dre:566841 dre:436718 dre:323883 dre:100004116 dre:100332300 dre:556634 dre:559723 |
| dre04920 | Adipocytokine signaling pathway | 4/900 | 108/8379 | 0.997975405380127 | 0.999573419006275 | 406473 100037357 564295\_2 100149042 | pck2 tnfrsf1b traf2a agrp | dre:406473 dre:100037357 dre:564295 dre:100149042 |
| dre00510 | N-Glycan biosynthesis | 1/900 | 68/8379 | 0.999573419006275 | 0.999573419006275 | 327601\_1 | alg8 | dre:327601 |
